# New tools for evaluating LQAS survey designs

**DOI:** 10.1186/1742-7622-11-2

**Published:** 2014-02-15

**Authors:** Lauren Hund

**Affiliations:** 1 Department of Family and Community Medicine, University of New Mexico, 2400 Tucker Avenue Northeast, Albuquerque NM 87131, USA

**Keywords:** Acceptance sampling, LQAS, Survey design

## Abstract

Lot Quality Assurance Sampling (LQAS) surveys have become increasingly popular in global health care applications. Incorporating Bayesian ideas into LQAS survey design, such as using reasonable prior beliefs about the distribution of an indicator, can improve the selection of design parameters and decision rules. In this paper, a joint frequentist and Bayesian framework is proposed for evaluating LQAS classification accuracy and informing survey design parameters. Simple software tools are provided for calculating the positive and negative predictive value of a design with respect to an underlying coverage distribution and the selected design parameters. These tools are illustrated using a data example from two consecutive LQAS surveys measuring Oral Rehydration Solution (ORS) preparation. Using the survey tools, the dependence of classification accuracy on benchmark selection and the width of the ‘grey region’ are clarified in the context of ORS preparation across seven supervision areas. Following the completion of an LQAS survey, estimation of the distribution of coverage across areas facilitates quantifying classification accuracy and can help guide intervention decisions.

## Introduction

Lot Quality Assurance Sampling (LQAS), also referred to as sampling for attributes and acceptance sampling, has a long history of applications in industrial quality control [1,2]. In the past 20 years, simple LQAS binary classification surveys have become increasingly popular in global health care applications [3]. In these LQAS surveys, an area is classified as having acceptable or unacceptable coverage of a health indicator by sampling from the region and counting the number of individuals with positive values of the indicator.

LQAS is a statistical tool based on frequentist notions of misclassification error. The development of generic training manuals has allowed survey designers to avoid the statistical principles behind LQAS, relying on cookbook formulas [4]. Subsequently, decision-making via LQAS in public health has been criticized [4-6]. Specific criticisms of LQAS surveys include difficulty in interpreting the results and high false positive rates [5,7]. To address these criticisms, Olives and Pagano (2010, 2013) illustrate that using a Bayesian approach facilitates quantifying the accuracy of LQAS classifications and illustrate how to apply Bayesian LQAS (B-LQAS) designs in public health applications [8,9]. Myatt and Bennett (2008) propose monitoring transmitted HIV drug resistance in developing countries use using sequential LQAS survey designs with Bayesian interpretations [10]. Applications of B-LQAS in public health have not been applied frequently in practice.

The idea of melding Bayesian and frequentist ideas to improve statistical inferences has been gaining in popularity, *e.g.*[11,12]. In LQAS surveys, no standard protocol or toolset exists for assessing implications of design parameter selection on the classification accuracy. Using reasonable prior beliefs about the distribution of coverage can inform and improve the selection of LQAS design parameters and help interpret survey results. This paper addresses merging Bayesian and frequentist ideas when designing LQAS surveys to provide perspective on LQAS classification accuracy. Tools for quantifying classification accuracy before and after the survey are proposed; corresponding software programs are provided for implementing these tools. After conducting the survey, the survey data can be aggregated to inform about the classification accuracy of the design. This paper is structured as follows. First, LQAS surveys (as often implemented in public health applications) are described; and data from two consecutive LQAS surveys in Nepal are introduced. Next, limitations to a wholly Bayesian or frequentist design procedure are discussed. To address these limitations, a simple step-by-step process that incorporates Bayesian and frequentist concepts is proposed for designing LQAS surveys. Finally, *post hoc* measures of classification accuracy are proposed using the collected survey data; and these methods are applied to assess the classification accuracy of the Nepal LQAS survey design.

## LQAS survey design

LQAS is a binary classification procedure for classifying the coverage of an indicator as acceptable or unacceptable within a supervision area (SA). In a classical LQAS survey, *n* individuals are randomly sampled from SA *i*. The number of successes *X*_*i*_ (based on the indicator) are counted among the *n* individuals. The SA coverage is classified as acceptable if *X*_*i*_>*d* and unacceptable if *X*_*i*_≤*d*. The key design question is how to select *n* and *d* such that the procedure has good classification properties.

The choice of *n* and *d* is determined by two equations that control the risk profile of the classification procedure:

(1) $$\begin{array}{l} P(X_{i}\leq d|n,p_{i}=p_{u})\;\;\leq \;\;\alpha \\ P(X_{i}>d|n,p_{i}=p_{l})\;\;\leq \;\;\beta , \end{array}$$

where *X*_*i*_∼*Binomial*(*n*,*p*_*i*_). The risk *α* is the probability of classifying an area as unacceptable when *p*_*i*_=*p*_*u*_. The risk *β* is the probability of classifying an area as acceptable when *p*_*i*_=*p*_*l*_. Areas with coverage between *p*_*l*_ and *p*_*u*_ are in the ‘grey region’. Misclassification risks are not explicitly restricted for areas in the grey region; that is, the classification procedure is not designed to accurately distinguish between areas with true coverages lying in the grey region.

The following steps are used to design an LQAS survey: 

1. Choose the binary indicator of interest and delineate the SAs.

2. Select upper and lower threshholds *p*_*l*_ and *p*_*u*_.

3. Select risks *α* and *β* corresponding to the thresholds in Step 2.

4. Iteratively solve for *n* and *d* in Equation 1 using the binomial cumulative mass function (typically with a software program).

The parameters *p*_*l*_,*p*_*u*_,*α*, and *β* are selected based on subject-matter knowledge, often using the following guidance: an SA with true coverage at or above *p*_*u*_ should be classified as unacceptable with low probability; and an SA with true coverage below *p*_*l*_ should be classified as acceptable with low probability. The risks *α* and *β* are the maximum allowable risks of misclassification at the upper threshold *p*_*u*_ and lower threshold *p*_*l*_, respectively. This guidance for parameter selection may be sub-optimal, especially when it is expected that a high proportion of SAs will have true coverage in the grey region, between *p*_*l*_ and *p*_*u*_.

### Example - ORS preparation in Nepal

Throughout this paper, the survey described in [13] and the data provided therein are referenced as an illustrative example. The survey and data, as described in [13], are briefly summarized. LQAS was used to monitor whether mothers correctly prepared of Oral Rehydration Solution (ORS) in 7 supervision areas (SAs) in Nepal [13]. A baseline survey was conducted in January 1999 to monitor the coverage of the indicator “correct ORS preparation”, and a follow up was conducted in January 2000. The goal of the January 2000 survey was to classify areas as achieving or failing to achieve the benchmark coverage target of 65%.

Within an SA, *n*=19 mothers were sampled, and *X*_*i*_ correctly prepared ORS. The decision rule *d* was selected, and, if *X*_*i*_>*d*, the SA was classified as achieving the benchmark; otherwise, the SA was classified as failing to achieve the benchmark. In the January 2000 follow-up survey, the authors selected a lower threshold *p*_*l*_ = 35% and an upper threshold *p*_*u*_ = 65%; misclassification risks *α* and *β* were restricted to less than 10%. The final sample size was *n*=19 and decision rule *d*=9. A subset of the survey results, as shown in [13], are reproduced in Table 1.

**Table 1:** Nepal ORS data from baseline (June 1999) and follow-up (January 2000)

**SA**	**June 1999**	**January 2000**
1	7	7
2	7	9
3	12	14
4	9	13
5	11	17
6	16	19
7	8	12
Average		
coverage	52.6%	68.2%

Number of mothers correctly preparing ORS out of 19 are displayed for each of the seven supervision areas [13].

### Comparing classical and Bayesian LQAS designs

One of the most common errors in statistical practice is the misinterpretation of the p-value [14]. In hypothesis testing, p-values are often incorrectly ascribed Bayesian interpretations. Specifically, p-values are often (incorrectly) interpreted as the probability that the null hypothesis is true, given the data (versus the probability of observing data as extreme as what was observed, given the null hypothesis). Hence, the conditioning event is incorrectly reversed.

While LQAS is not explicitly a hypothesis testing procedure, a similar error frequently occurs in LQAS applications: frequentist classification risks *α* and *β* are ascribed Bayesian interpretations. This error occurs because Bayesian risks are typically more informative in decision-making [5,8]. For example, classical LQAS risks pertain to the probability of classifying an SA as achieving the benchmark (or failing to reach benchmark achievement), given the true coverage probability (Equation 1). The probability that coverage truly exceeds (or does not exceed) the benchmark, given a classification of benchmark achievement, is typically a more interesting quantity for guiding decision making. Consequently, the risks *α* and *β* are often incorrectly interpreted in this manner.

To address the fact that Bayesian risks are more informative for decision making, Olives and Pagano (2009) proposed using a Bayesian classification procedure (B-LQAS). The fundamental differences between Bayesian and classical LQAS designs are the reversal of the conditioning event and the conceptualization of *p*_*i*_ as a random variable in Bayesian surveys (Table 2). In the B-LQAS design, the upper and lower thresholds, *p*_*l*_ and *p*_*u*_, are again specified. Rather than specifying frequentist classification risks *α* and *β*, the authors use Bayesian classification risks; namely, *α*_*B*_ is the probability that *p*_*i*_>*p*_*u*_, given that *X*_*i*_≤*d*, and *β*_*B*_ is the probability that *p*_*i*_<*p*_*l*_ given that *X*_*i*_>*d*[8]. The Bayesian risks *α*_*B*_ and *β*_*B*_ are conditional on the classification decision. To calculate these classification risks, Bayesian designs require specification of one additional quantity, a prior distribution $\hat{\pi }()$. The specified prior distribution $\hat{\pi }()$ is an estimate of the distribution of *p*_*i*_, denoted *π*(). Heuristically, in a Bayesian framework, coverage *p*_*i*_ is a random variable that fluctuates, and *π*() measures the range of feasible variability in *p*_*i*_ at the time of the survey.

**Table 2:** Reversal of the conditioning even in Bayesian and frequentist LQAS surveys

**Classical**		**Bayesian**
*α*=*P*(Fail to achieve		*α*_*B*_=*P*(*p*_*i*_≥*p*_*u*_|Fail to achieve
benchmark|*p*_*i*_=*p*_*u*_)		benchmark)
*β*=*P*(Achieve benchmark|*p*_*i*_=*p*_*l*_)		*β*_*B*_=*P*(*p*_*i*_≤*p*_*l*_|Achieve
		benchmark)

Conceptualization, and subsequently, estimation of this distribution *π*() is a difficult task. In industrial quantity control, a precise estimate of *π*() can be constructed by measuring the defect rate for a batch of goods (for, say, a production line) across many different batches. In public health, conceptualizing this prior distribution is less straightforward, because coverage rates fluctuate over time and space; hence, *π*() is never known prior to the survey. One possible definition of *π*() is the underlying distribution of coverage across SAs at the time of the survey. This definition implicitly assumes that the region contains a large number of SAs; that SAs are independent; and that no prior knowledge about differences in coverage by SA exists. This definition of *π*() is used throughout the rest of this manuscript.

Often, surveys are conducted to update knowledge about *π*(), which likely changes over time [9]. For instance, in the Nepal ORS coverage example, efforts are made to improve coverage over time; understanding temporal changes in coverage (*i.e.* temporal changes in *π*()) is a goal of the LQAS surveillance program. B-LQAS survey designs rely on correct *a priori* specification of this distribution, namely $\hat{\pi }()=\pi ()$, and are sensitive to the choice of $\hat{\pi }()$[9]. Hence, the major limitation of Bayesian designs is the requirement of correct prior specification (which is unlikely in practice).

Misspecification of *π*() in the design phase biases inferences, with the severity of bias depending on the degree of misspecification. To understand the root of this bias, note that the risks *α*_*B*_ and *β*_*B*_ for a classification procedure are a function of the specified prior $\hat{\pi }()$; the collected data naturally does not inform the survey design or decision rules, whereas the prior selection does. This prior $\hat{\pi }()$ (the estimate of the underlying distribution of coverage in the population) is not utilized in the same manner as standard Bayesian analyses, where prior information is updated using collected data to construct a posterior distribution. For example, in Bayesian statistics, when little prior information is available, non-informative priors are chosen to reflect the lack of prior beliefs (allowing the data to dominate prior beliefs). In B-LQAS designs, non-informative priors are actually informative. A non-informative (flat) prior would suggest that all values of *p*_*i*_ are *a priori* equally likely, which is often a strong, and incorrect, assumption. The prior $\hat{\pi }()$ is specified before the survey occurs, and classification rules depend on the distribution $\hat{\pi }()$. Hence, the use of the term “prior” for $\hat{\pi }()$ is an unfortunate misnomer for B-LQAS designs.

If the specified prior does not reflect the distribution of coverage at the time of the survey, the risks *α*_*B*_ and *β*_*B*_ do not represent the true error rates for the classification procedure. Further, there is no way to *a priori* assess the accuracy of the prior $\hat{\pi }()$; the only way to assess the accuracy of the prior is through a *post hoc* estimation of the prior from the collected survey data.

### Design tools for an LQAS survey

LQAS can be conceptualized as a population screening tool, where SAs are screened to examine if a benchmark coverage level is achieved. For standard screening tools (or diagnostic tests), sensitivity and specificity measure the true positive and negative rates of screening tools; namely, these quantities answer the question “conditional on disease status, how often does the screening tool give the correct diagnosis?” As a patient who tests positive or negative based on the screening tool, the sensitivity and specificity are not relevant. Rather, positive predictive value (PPV) and negative predictive value (NPV), which quantify the probability of correct diagnosis conditional on the test result, help the patient understand the likelihood of having the disease. PPV and NPV are typically calculated from sensitivity and specificity, using Bayes theorem and knowledge of the population prevalence.

In classical LQAS surveys, risks *α* and *β* condition on the true population coverage, analogous to sensitivity and specificity which condition on disease status. Classical LQAS survey designs always maintain the specified classification risks *α* and *β*, regardless of the underlying coverage distribution *π*(). In classical LQAS surveys, operating characteristic (OC) curve and risk curves [9] are often plotted to summarize the design properties. The OC curve is defined as *P*(*X*_*i*_>*d*|*p*); and the risk curve is defined as *P*(incorrect classification|*p*), where classification is incorrect if *X*_*i*_>*d*|*p*<*p*^∗^ or *X*_*i*_≤*d*|*p*>*p*^∗^. The risks *α* and *β*, the OC curve, and the risk curve are frequentist design summaries that condition on the true coverage, and therefore do not directly inform classification accuracy. That is, these measures do not inform how likely it is that an SA has achieved the benchmark, *conditional on the classification decision* (analogous to PPV and NPV); this measure is a function of the distribution of coverage, *π*().

Classification accuracy of a survey design measures how frequently the design correctly classifies coverage and pertains to PPV and NPV, which are inherently Bayesian quantities. In order to define the PPV and NPV of a design, it is useful to first designate a programmatic target *p*^∗^, denoting the cut-off for correct versus incorrect classifications [4]. In the Nepal example, selecting *p*^∗^=0.65 is a reasonable choice; with *p*^∗^=0.65, classifying areas with true coverage in the grey region (35%-65%) as achieving the benchmark of 65% coverage is an error. Specification of *p*^∗^ is not mandatory for designing a survey, but is essential for evaluating the classification accuracy of the survey.

Throughout this article, the following definitions of PPV and NPV are used:

(2) $$\begin{array}{ll} \text{PPV} & =P(\text{high coverage}|\text{classified as high})\\ & =P(p_{i}>p^{*}|X_{i}>d)=P(X_{i}>d|p_{i}>p^{*})\\ & \;\times P(p_{i}>p^{*})/P(X_{i}>d)\\ & =P(X_{i}\;>\;d|p_{i}\;>\;p^{*})\;\;\int_{p^{*}}^{1}\;\;\pi (p)\text{dp}/\;\;\int_{p^{*}}^{1}\;\;P(X_{i}\;>\;d|p)\pi (p)\text{dp}\\ \text{NPV} & =P(\text{low coverage}|\text{classified as low})\\ & =P(p_{i}<p^{*}|X_{i}\leq d)=P(X_{i}\leq d|p_{i}<p^{*})\\ & \;\times P(p_{i}<p^{*})/P(X_{i}\leq d)\\ & =P(X_{i}\;\leq \;d|p_{i}\;<\;p^{*})\;\;\int_{0}^{p^{*}}\;\;\;\pi (p)\text{dp}/\;\;\int_{0}^{p^{*}}\;\;\;P(X_{i}\;\leq \;d|p)\pi (p)\text{dp} \end{array}$$

PPV and NPV are based on the unknown true underlying distribution for *p*_*i*_, *π*(). Estimates of these quantities, denoted $\hat{\text{PPV}}$ and $\hat{\text{NPV}}$, are calculated with respect to the specified prior distribution by substituting $\hat{\pi }()$ for *π*() into Equation 2. While *π*() will not be known explicitly for public health applications, a range of potential distributions could likely be elicited from program managers before conducting a survey (*i.e.* specify various different values of $\hat{\pi }()$ by considering feasible ranges for *p*_*i*_).

The proposed design tools estimate the classification accuracy (PPV and NPV) of a design for a range of distributions $\left\{\hat{\pi }()\right\}$. Simple calculations of $\hat{\text{PPV}}$ and $\hat{\text{NPV}}$ for various specifications of $\hat{\pi }()$ provide a sensitivity analysis for the classification accuracy of the survey design. For instance, if most SAs have true coverage in the grey region, the survey will either have very poor PPV or NPV. When selecting prior distributions, $\hat{\pi }()$ should be chosen to reflect current beliefs as closely as possible; using multiple plausible values of $\hat{\pi }()$ is best unless the actual value of *π*() is known with some certainty. Given that surveys are often conducted to measure changes in the distribution of coverage over time, substantial uncertainty will usually exist *a priori* in estimates of *π*().

The steps for designing a classical LQAS survey were described in the above section. The design parameters (*p*_*u*_, *p*_*l*_, *p*^∗^, *α* and *β*) can be selected by evaluating the classification accuracy of the design. Specifically, to select these design parameters, the following steps are proposed:

1. Select a programmatic target *p*^∗^. Then, select *p*_*l*_, *p*_*u*_, *α*, and *β*, using subject-matter knowledge and keeping *p*^∗^ in mind.

2. Determine *n* and *d* corresponding to this choice.

3. Plot risk curve [9] for the survey design.

4. Construct multiple plausible estimates of *π*(), using subject-matter knowledge and historical data. Calculate *P*(*p*<*p*^∗^) and *P*(*p*_*l*_<*p*<*p*_*u*_) for the distribution, to gauge how much of the mass of the prior lies above/below the target *p*^∗^ and within the grey region (*p*_*l*_,*p*_*u*_).

5. Calculate the PPV and NPV for the each specified prior $\hat{\pi }()$. Also, calculate the probability of true coverage lying within the grey region, given the classification.

6. Return to step (1) if the design parameters do not provide sufficiently accurate classifications; consider reducing the misclassification risks or narrowing the grey region.

The R package, lqasdesign, written in R version 2.15.2 [15], contains functions for designing LQAS surveys and evaluating the designs (the R package is Additional file 1). The package includes functions for calculating the sample size and decision rule for an LQAS design (Step 2 above); and functions for conducting sensitivity analyses to examine the design parameter choices for different prior specifications (Steps 2-4). To facilitate use of these tools, the package contains an interactive web-application for survey design, constructed using the shiny package from Rstudio [16]. Screen-shots and simple instructions for using the application are in Appendix B in Additional file 2. Instructions for using the package are in the package manual, accessed by typing vignette(~manual~,package= ~lqasdesign~) in R.

Eliciting various prior distributions in step (4) is non-trivial. Restricting to families of distributions with support between 0 and 1 is preferable when modeling proportions. The unimodal Beta distribution is implemented in the R package for simplicity. The Beta distribution is characterized by two parameters, *a* and *b*, and is denoted *B*(*a*,*b*). The mean of a *B*(*a*,*b*) distribution is *a*/(*a*+*b*), and the standard deviation is also a function of *a* and *b*. More properties of the Beta distribution are discussed in Appendix A in Additional file 2. Expanding the R functions to accommodate other prior distributions, such as a mixture of Beta distributions, is of interest in future work.

Using the provided R programs, the user can specify a mean and standard deviation for *p*_*i*_ to obtain a Beta prior. The Beta distribution is asymmetrical and can be highly skewed (making the standard deviation more difficult to specify). The R package contains functions for plotting the selected prior and calculating the probabilities in Step 4 of the design algorithm, to ensure that the user’s prior beliefs adequately match the shape of the selected distribution. The user can also input data from past surveys (across multiple SAs) and find the best-fitting Beta distribution for the data. The selected prior(s) should represent the range of current beliefs about the distribution of coverage in a region, specified using existing data, expert opinions, or both. In the section below, a step-by-step example of choosing various prior distributions using baseline data is considered.

### Application: ORS coverage survey design properties

**Figure 1:**
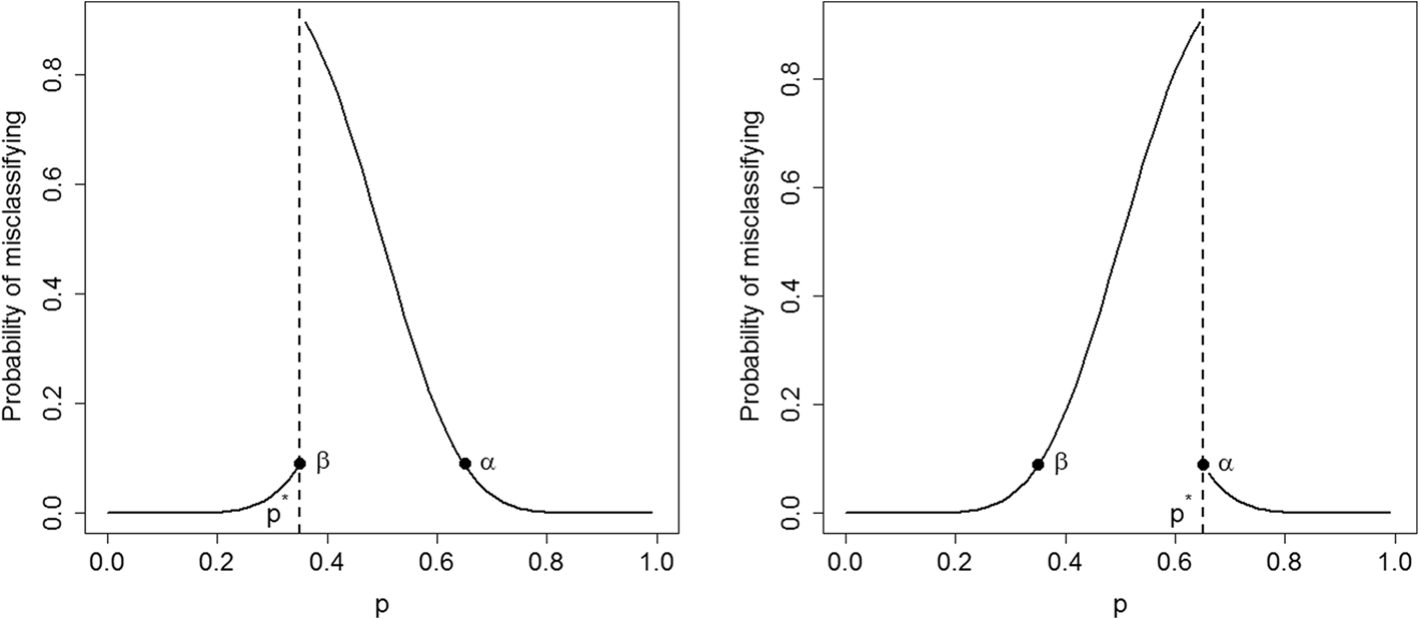
**Risk curves.** Risk curves for an LQAS design with *n*=19 and *d*=9, for *p*^∗^=0.35 and *p*^∗^=0.65

The evaluation tools are illustrated using the design of the January 2000 follow-up Nepal ORS coverage survey, with *n*=19 and *d*=9. The classification risks *α* and *β* are both 0.087. Hence, the probability of failing to achieve the benchmark when *p*>*p*_*u*_ is less than 0.087; and the probability of achieving the benchmark when *p*<*p*_*l*_ is less than 0.087. The risk curves with *p*^∗^=0.35 and *p*^∗^=0.65 are plotted in Figure 1.

**Figure 2:**
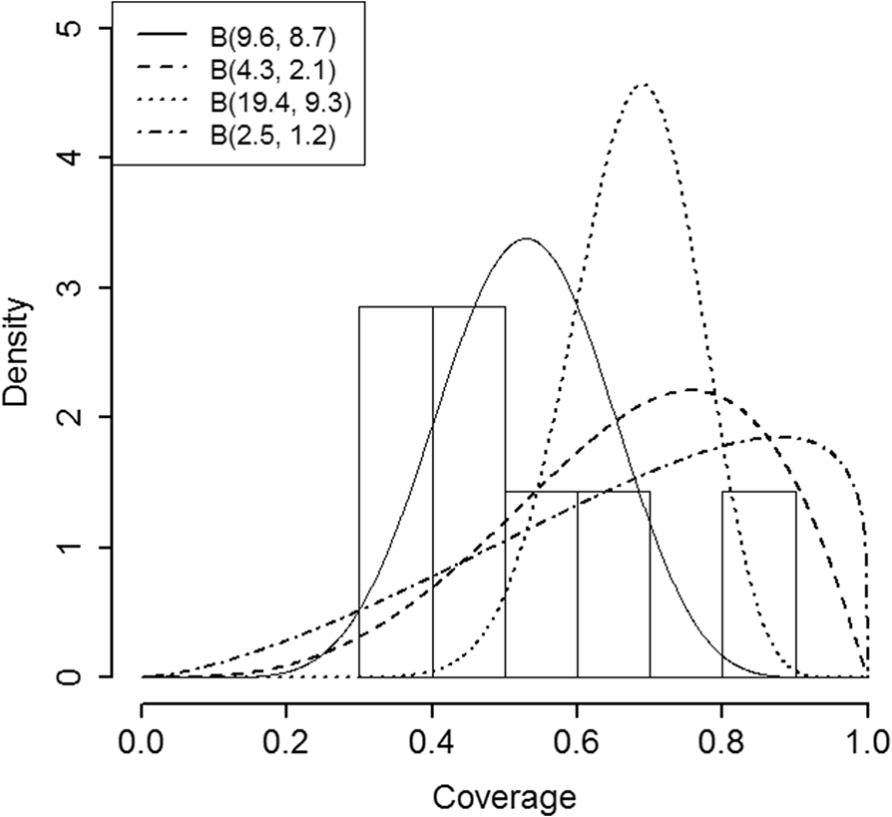
**Prior Distributions.** Prior distributions used in the ORS survey design sensitivity analysis. The histogram represents a plot of the actual data across the 7 SAs in January 1999.

Next, Bayesian summary measures for the survey design are examined, after specifying an underlying coverage distribution for *p*_*i*_. Several different $\hat{\pi }()$ distributions are considered: *B*(1,1), a flat, “non-informative” prior (coverage of an SA has an equal probability of taking on any value between 0 and 1); *B*(9.6,8.7), a Beta distribution consistent with the information observed at the first survey in January 1999; and *B*(4.3,2.1), a Beta prior consistent with the idea that mean coverage shifted by 15% from January 1999 to January 2000, but the standard deviation remained the same. PPV and NPV are sensitive to both the mean and variance of *π*(). Two additional priors are considered, choosing the same mean as the *B*(4.3,2.1) prior, but reducing the standard deviation by half (*B*(19.4,9.3)) and raising the standard deviation by 25% (*B*(2.5,1.2)) to assess sensitivity of the design properties to the spread of the distribution. Heuristically, the survey properties will be different if the *p*_*i*_s are constrained to a narrow range. These prior distributions are plotted in Figure 2.

Positive predictive value and negative predictive value are calculated for the design, assuming that *p*_*i*_ is a random variable generated from the prior distribution. Programmatic thresholds *p*^∗^=0.35 and *p*^∗^=0.65 are considered; the probability that true coverage lies in the grey region (*p*_*l*_ to *p*_*u*_) is also calculated. Results are displayed in Table 3.

**Table 3:** Properties of the survey designs for various prior specifications

	***p***^**∗**^**=** ***.*** **35**			***p***^**∗**^**=** ***.*** **65**			***p*** **∈(*.*** **35,** ***.*** **65)**		
***π*** **()**	***S*** **(*p***^**∗**^**)**	**PPV**	**NPV**	***S*** **(*p***^**∗**^**)**	**PPV**	**NPV**	***P***_***grey***_	**PPV**	**NPV**
B(1,1)	0.650	0.991	0.692	0.350	0.692	0.991	0.300	0.300	0.300
B(9.6, 8.7)	0.937	0.995	0.139	0.143	0.243	0.986	0.794	0.752	0.848
B(4.3, 2.1)	0.957	0.998	0.213	0.593	0.728	0.956	0.363	0.270	0.743
B(19.4, 9.3)	1.000	1.000	0.002	0.634	0.688	0.832	0.366	0.312	0.831
B(2.5, 1.2)	0.908	0.997	0.381	0.592	0.766	0.972	0.316	0.231	0.592

The table quantities with respect to p^*^ are defined as: *S*(p^*^) =*P*(*p* >p^*^), *PPV* =*P*(*p* >p^*^ | *X*_*i*_ > 9) and *NPV* =*P*(*p* <p^*^ |*X*_*i*_ ≤ 9). For the grey region, *P*_*grey*_ =*P*(*p* ∈{.35,.65 }), *PPV* =*P*(*p*_*l*_ <*p* <*p*_*u*_ | *X*_*i*_ > 9) and *NPV* =*P*(*p*_*l*_ <*p* <*p*_*u*_| *X*_*i*_ ≤ 9).

These calculations help clarify role of *p*^∗^ in interpreting LQAS surveys. Choosing *p*^∗^=.65, the survey has excellent NPV and mediocre PPV. Hence, areas classified as failing to achieve the benchmark likely have coverage less than 65%. Areas achieving the benchmark may or may not have coverage greater than 65%. Given that *p*^∗^=65*%* is likely the most contextually relevant threshold for the application, program managers would know to interpret ‘benchmark achievement’ with caution or to construct a different design by changing the thresholds *p*_*l*_ and *p*_*u*_. If instead the benchmark were *p*^∗^=.35, the survey has excellent PPV and mediocre to poor NPV, depending on the prior. In this case, if an area is classified as achieving the benchmark, it is likely that coverage is at least 35%. If an area is classified as failing to achieve the benchmark, it is unclear whether coverage is greater than or less than 35%.

Lastly, examining the grey region properties clarifies that the probability of an area having true coverage in the grey region is non-negligible for all of the prior selections. This result is not surprising, because the grey region spans 30% of the support of *p*_*i*_. Narrowing the grey region would improve classification accuracy at the cost of an increased sample size.

Appendix C in Additional file 2 contains R code for replicating all of the analyses in this manuscript.

## Post-survey tools for LQAS surveys

LQAS data are often summarized by presenting a confidence interval for coverage, aggregating over all SAs; this confidence interval provides a measure of uncertainty associated with the overall coverage in the region. Additionally, the number of SAs classified as acceptable/unacceptable is usually presented. These standard summary measures do not inform the classification accuracy of the design. Understanding classification accuracy of the design procedure can help determine how to allocate resources. Estimation of the coverage distribution *π*() following the survey can help to *a posteriori* measure the accuracy of the classifications.

As an example, hypothetically suppose that, out of 10 SAs, 5 achieve the benchmark and 5 do not. Consider two different extreme scenarios: 1) the coverage distribution *π*() is bimodal, and 0 areas have true coverage between 35% and 65%; and 2) *π*() is unimodal and all 10 areas have true coverage between 35% and 65%. Using a standard LQAS survey protocol, it is unclear how to distinguish between scenario 1 or 2 for decision-making. For surveys like the Nepal survey, with a grey region spanning almost a third of the support of *p*_*i*_, scenario 2 is likely common. Characterizing the distribution of coverage across SAs, *π*(), and incorporating this information into the decision-making process can improve the efficacy of LQAS as a monitoring and evaluation tool.

When LQAS surveys are conducted in many SAs within a region, the underlying distribution of coverage across SAs in the region, *π*(), can be estimated and used to calculate the expected proportion of SAs with coverage below *p*^∗^ and with coverage in the grey region, *p*_*l*_ to *p*_*u*_. To estimate *π*(), again assume that the true prevalence in an SA, *p*_*i*_, is a random variable drawn from *π*().

The distribution *π*() is estimated using several approaches: assuming a parametric Beta distribution; using a simple non-parametric histogram; and using kernel density estimation [17,18] for non-parametric smoothing. For a review on density estimation, see [19] and references within. For the kernel density estimator, the default bandwidth *h*∗*m*^−.3^ is used in the R program and throughout the analysis, where *h* is the bandwidth using Silverman’s rule of thumb [20] and *m* is the number of SAs. This bandwidth is selected to prioritize unbiasedness (over variance reduction) in estimation and avoid oversmoothing [21,22]. Following density estimation, the probabilities *P*(*p*_*i*_<*p*^∗^) and *P*(*p*_*l*_<*p*_*i*_<*p*_*u*_) are estimated, with corresponding standard errors estimated using bootstrap resampling [23].

By estimating *π*() from the data, program managers can learn important properties about the distribution of *p*_*i*_ to inform intervention decisions. A bimodal distribution implies that some areas are performing well, while others are performing poorly. A unimodal distribution centered in the grey region suggests that area-specific interventions might not be as effective as a region-wide intervention, and binary classifications should not be over-interpreted. Further, the estimated density can guide survey design in the next round of surveillance. In the Nepal survey design application, data from the first round of surveillance were used to construct an array of prior distributions, first estimating the density from baseline data and then shifting the mean and varying the standard deviation of this estimated density.

When the number of subjects sampled per SA and number of SAs are both large, the nonparametric density estimators are unbiased, and the parametric estimator is unbiased if the Beta model is correct. In finite samples, density estimators are biased. In Appendix D in Additional file 2, finite sample bias and standard errors are evaluated for *P*(*p*_*i*_<*p*_*l*_), *P*(*p*_*i*_>*p*_*u*_), and *P*(*p*_*l*_<*p*_*i*_<*p*_*u*_) using a simulation study. The finite sample bias is non-negligible and varies depending on the mode of estimation. Estimating the probabilities *P*(*p*_*i*_<*p*_*l*_), *P*(*p*_*i*_>*p*_*u*_), and *P*(*p*_*l*_<*p*_*i*_<*p*_*u*_) can inform classification accuracy, but the estimated probabilities and standard errors may exhibit substantial bias in small sample sizes.

### Application: ORS coverage density

**Figure 3:**
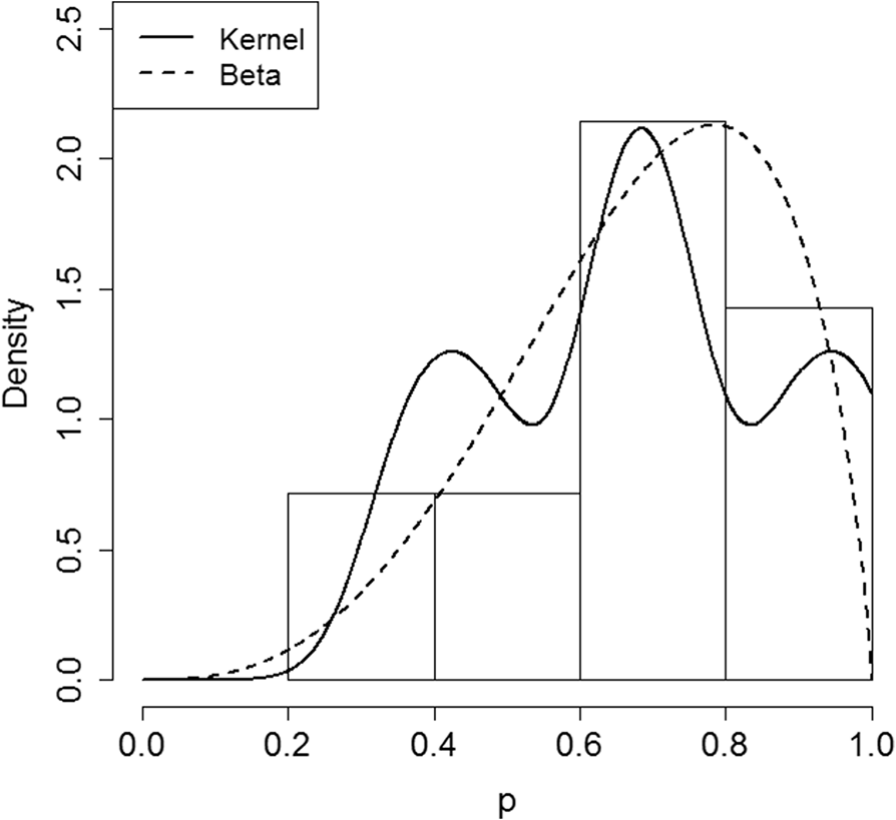
**Density Estimates.** Underlying coverage density estimates following data collection in the 7 SAs in January 2000.

The survey conducted in January 2000 contained only 7 SAs and 19 people per SA. Therefore, all of the proposed density estimators are biased, and it is important to avoid over-interpreting these results. The estimates of *π*() using the parametric Beta distribution, the kernel density estimator, and the crude histogram are plotted in Figure 3.

The estimated percent of areas with true coverage in the grey region is 42.9% (sd = 18.6%) using the crude histogram; 36.9% (sd =14.0%) using kernel density estimation; and 34.2% using the Beta distribution (standard error not available due to small sample size and lack of estimator convergence in bootstrap samples). While these estimates are likely somewhat biased, the results suggest that a high proportion of SAs could have true coverages in the grey region.

## Conclusion

In this paper, a simple evaluation framework for LQAS survey designs is developed by melding Bayesian and frequentist ideas. The suggested tools are implemented within the free software program R; detailed instructions and a user-friendly web-based application should facilitate the use of these tools. The practicality of LQAS lies in its simplicity; the entire design is determined by four parameters: *p*_*l*_,*p*_*u*_,*α*, and *β*, and can be evaluated with respect to a programmatic target *p*^∗^. However, arbitrary specification of the design parameters without considering concepts such as positive and negative predictive value is potentially dangerous. The implications of choosing a grey region of width 30% are clearer following these calculations. When design properties are less than ideal, abandoning binary classification in favor of a three-tiered [24] or double sampling approach [25] is a viable option.

In this paper, Bayesian survey designs are discussed based on the classification risks *α*_*B*_ and *β*_*B*_, to facilitate contrasting the Bayesian and classical survey designs. Alternative Bayesian designs (*e.g.* using different loss functions) are discussed in [8,9]. Due to the subjective specification of the prior and potential for bias, purely Bayesian designs can perform poorly in practice unless the prior is known with certainty.

Public health applications of LQAS typically use simple binary classification for decision-making, though other, non-binary types of outcomes have been explored in public health. Olives *et. al* (2012) construct classification designs for ordinal variables with more than two categories [24]. Hypergeometric models are also used in practice when population sizes are small *e.g.*[25,26]. Future work should explore developing LQAS design diagnostic tools for these different outcome models, with appropriate prior selection; as well as explore developing LQAS designs and analysis tools for other types of outcomes, such as the Poisson model for rates and the normal model for means.

This paper is intended as a first step toward developing more sophisticated tools for LQAS survey design evaluation using Bayesian concepts. LQAS designs are being extended for more complex applications [10,27,28]. As the complexity of these surveys increases, training materials and additional survey evaluation tools that encourage program managers to understand the entire LQAS probabilistic framework will become increasingly valuable.

## Competing interests

The authors declare that they have no competing interests.

## Supplementary Material

Additional file 1 **lqasdesign R package.** Additional file 2 contains the lqasdesign R package. Click here for file

Additional file 2 **Appendices.** Appendix A contains a description of the Beta distribution; Appendix B contains a description of the web-based R application; Appendix C contains complete R code for reproducing the analysis in the manuscript; and Appendix D contain a simulation study assessing properties of the density estimators. Click here for file
