# Supplementary material for: New tools for evaluating LQAS survey designs
Source: Emerg Themes Epidemiol. 2014 Feb 15;11:2. doi: 10.1186/1742-7622-11-2 (PMC3931287; doi:10.1186/1742-7622-11-2)
Supplement: Additional file 1 — lqasdesign R package. Additional file 2 contains the lqasdesign R package. [file 1742-7622-11-2-S1.zip › lqasdesign/doc/index.html]

R: Vignettes

# Vignettes

---

## Vignettes from package 'lqasdesign'

| lqasdesign::manual |  | User manual | PDF | source |  |
