# Supplementary material for: New tools for evaluating LQAS survey designs
Source: Emerg Themes Epidemiol. 2014 Feb 15;11:2. doi: 10.1186/1742-7622-11-2 (PMC3931287; doi:10.1186/1742-7622-11-2)
Supplement: Additional file 1 — lqasdesign R package. Additional file 2 contains the lqasdesign R package. [file 1742-7622-11-2-S1.zip › lqasdesign/doc/manual.pdf]

Using the lqasdesign R package

Lauren Hund

November 19, 2013

Contents

1 Summary **2**

2 Basic Commands for Classical LQAS Survey **2**
2.1 Overview of Classical Lot Quality Assurance Sampling 2
2.2 Using the web-based survey design application 3
2.3 Designing a survey using `lqasdesign` 3

3 Design Evaluation Tools **4**
3.1 Specifying a prior 5
3.2 Calculating survey properties 6
3.3 Post-survey tools 7

4 Finite population surveys - the hypergeometric distribution **8**

5 Cluster LQAS survey designs **10**
5.1 Incorporating uncertainty in p^* into the design 13

6 Double Sampling **14**

7 Figures **17**

1 Summary

In this vignette, we illustrate how to use the package `lqasdesign` to design an LQAS survey, following [4]. The package includes survey design functions, as well as functions to evaluate the designs.

2 Basic Commands for Classical LQAS Survey

2.1 Overview of Classical Lot Quality Assurance Sampling

We are interested in the coverage p in a supervision area (SA) within a region. We collect data on n individuals and observe X successes. Then, $X \sim Binomial(n, p)$. If $X \geq d$, we classify the region as having high coverage; otherwise, we classify coverage as low.

The risk of the classification procedure is determined by the equations:

$$\begin{aligned} P(X \leq d|n, p = p_u) &\leq \alpha \\ P(X > d|n, p = p_l) &\leq \beta \end{aligned} \quad (1)$$

Within this framework, α is the probability of classifying an SA as low coverage when coverage $p = p_u$; and β is the probability of classifying an SA as high coverage when coverage $p = p_l$. The user must specify the design parameters p_l, p_u, α, and β.

The OC curve the probability of low classification as a function of the population coverage, given a sample size n and decision rule d:

$$OC(p) = P(X \leq d|n, p). \quad (2)$$

Another function of the population coverage is the risk curve [1], defined as

$$R(p) = P(\text{classification error}|n, p, p^*). \quad (3)$$

When plotting the risk curve, we specify an additional parameter, p^*, to demarcate the programmatic target, defined as the exact cut-off at which an area should be declared high or low. If we do not specify p^*, then the risk curve is undefined between p_l and p_u.

If we conceptualize p as a random variable, then, for a given prior/underlying distribution $\pi()$, we define the sensivitiy, specificity, positive predictive value (PPV), and negative predictive value (NPV) as:

$$\begin{aligned} Sens &= P(X > d|p > p^*) \\ Spec &= P(X \leq d|p < p^*) \\ PPV &= P(p > p^*|X > d) \\ NPV &= P(p < p^*|X \leq d) \end{aligned}$$

2.2 Using the web-based survey design application

The interactive web-based application allows users to interactively change the survey design parameters [5]. To use the application, simple type `designsurvey()`.

For help choosing the Beta prior values for the survey evaluation, type `surveyprior()`, which loads an additional web-based application.

2.3 Designing a survey using `lqasdesign`

First, we illustrate how to design a basic LQAS survey. The `lqas` command allows the user to input p_l, p_u, α, and β, and returns the smallest sample size and corresponding decision rule that satisfy the constraints in Equation 1.

We select $p_l = 0.35, p_u = 0.65, \alpha = 0.09, \beta = 0.09$ and run the command:

```
design <- lqas(pl = 0.35, pu = 0.65, alpha = 0.09, beta=0.09)
```

We have constructed an object of class `lqas` called `design`. We can now apply several different functions to this object, to examine the properties of our survey design. First, we use the `summary` command to view the basic properties of the design.

```
> summary(design)
The LQAS design parameters are:

Sample Size: 19
Decision Rule: 9

Classify as high if X > d.

The true error levels for this design are:
 alpha = 0.0875
  beta = 0.0875

Design Parameters:
pl =  0.35 and pu =  0.65
alpha =  0.09 and beta =  0.09
Probabilities were calculated using the binomial distribution.
```

To constrain α and β errors to below 0.09, we collect a simple random sample of 19 individuals. We will classify the coverage as high if $X > 9$. Due to the discrete nature of the binomial distribution, the true α and β errors are 0.0875.

To plot the OC curve corresponding to the design, defined in Equation 2, we type:

```
plot(design)
```

which returns the graph in Figure 1.

To plot the risk curve corresponding to the design, defined in Equation 3, we type:

```
plot(design, risk=T)
```

which returns the graph in Figure 2.

Alternatively, if we have an absolute cut-off, say $p^* = 65\%$, for demarcating low from high classifications, we can type:

```
plot(design, risk=T, pstar=0.65)
```

which returns the graph in Figure 3.

Rather than plotting the OC curve, if we simply want to obtain the OC probabilities, we use the `oc` function.

```
> oc(design, p = c(.25, .35, .5, .65, .75))
     p      oc
1 0.25 0.99502
2 0.35 0.92981
3 0.50 0.50000
4 0.65 0.10053
5 0.75 0.00498
```

In this function, we can also specify the limits for the OC probabilities with a corresponding increment.

```
> oc(design, plim = c(0.25, 0.75), by = 0.1)
```

Alternatively, we could simply use the default settings, by typing

```
oc(design)
```

The commands above are useful for designing a basic LQAS survey and summarizing the basic properties of the design.

3 Design Evaluation Tools

After choosing a decision rule d and sample size n, we recommend conducting a sensitivity analysis by examining the PPV and NPV of the design with respect to several different priors, $\pi()$. The prior, or underlying coverage distribution, is the distribution of p across SAs.

3.1 Specifying a prior

The `makeprior` function allows the user to specify a Beta prior using historical information and/or the mean and standard deviation of the distribution.

For instance, we can construct a Beta prior using results from previous surveys with sample size 19 at 7 SAs:

```
> results <- c(7, 7, 12, 9, 11, 16, 8)
> ss <- 19
> prior <- makeprior(X=results, n=ss)
> summary(prior)
Coverage follows a beta distribution with parameters:
alpha: 9.658703
beta: 8.692833

This distribution has mean 0.5263158 and standard deviation 0.171894 .

Prior was calculated using  7 previous survey results.
```

The function uses method of moments (default) or maximum likelihood to estimate the prior density; both methods are biased in finite samples. Therefore, this estimated distribution should not be interpreted as the precise prior distribution for p when the number of SAs is small or the sample size per SA is small (both are true in this instance). However, the function provides a good starting point for prior specification during the sensitivity analysis when previous data is available. To examine the histogram of the estimates of p versus the fitted prior distribution, use the plot function:

```
plot(prior)
```

which returns the plot in Figure 4.

We can also combine previous results with historical information about the mean and standard deviation of the prior:

```
> prior2 <- makeprior(X=results, n=ss, mean=0.4, sd = 0.1, weight = 0.5)
> summary(prior2)
Coverage follows a beta distribution with parameters:
alpha: 6.056644
beta: 7.020201

This distribution has mean 0.4631579 and standard deviation 0.1329031 .

Prior was calculated using  7 previous survey results.
```

```
Previous surveys received weight 0.5,
 and historical prior information received weight 0.5.
```

Lastly, we could specify the mean and standard deviation of the distribution, without incorporating any data:

```
> prior3 <- makeprior(mean=0.4, sd = 0.1)
> summary(prior3)
```

3.2 Calculating survey properties

After specifying a prior, we use the `designeval` function to evaluate the design with respect to the prior and a progammatic threshold p^*.

```
> eval1 <- designeval(n=19, d=9, pstar=.65, prior=c(prior$alpha, prior$beta))
> summary(eval1)
Assuming coverage has an underlying Beta(9.658703,8.692833) distribution,
P(p < pstar)=  0.8566
P(p > pstar)=  0.1434

Using a sample of size 19 and declaring high if X > 9 ,
the survey design has the following properties for a cutoff of 0.65:

Sensitivity - P(high | p > pstar)              0.9599
Specificity - P(low | p < pstar)               0.4991
Positive Predictive value - P(p > pstar | high) 0.2431
Negative Predictive value - P(p < pstar | low)  0.9859
```

This function provides several important pieces of information. First, $P(p < p^*)$ informs the user of where the mass of the prior lies, relative to the threshold p^*. Next, the sensitivity, specificity, PPV, and NPV are provided, relative to p^*. PPV and NPV are usually the quantities of interest in determining the accuracy of the design, but we include sensitivity and specificity for thoroughness.

If we do not specify a programmatic threshold p^* and instead provide p_l and p_u, the function returns the survey properties with respect to p_l and p_u, which may be of interest. The function summarizes the proportion of areas with coverages in the grey region, both unconditionally, $P(p_u < p < p_l)$, and conditional on classification, $P(p_l < p < p_u|X > d)$ and $P(p_l < p < p_u|X \leq d)$.

```
> eval2 <- designeval(n=19, d=9, pl=.35, pu=.65, prior=c(prior$alpha, prior$beta))
> summary(eval2)
Assuming coverage has an underlying Beta(9.658703,8.692833) distribution,
```

```
P(p < pl)       =  0.063
P(pu < p < pl) =  0.7936
P(p > u)       =  0.1434

Using a sample of size 19 and declaring high if X > 9 ,
the survey design has the following properties for
 lower and upper thresholds 0.35 and 0.65:

Sensitivity - P(high | p > pu)               0.9599
Specificity - P(low | p < pl)                0.9553
Positive Predictive value - P(p > pu | high) 0.2431
Negative Predictive value - P(p < pl | low)  0.1388

Grey region properties:

P(high | pl < p < pu) 0.5368
P(low |  pl < p < pu) 0.4632
P(pl < p < pu | high) 0.7521
P(pl < p < pu | low)  0.8477
```

3.3 Post-survey tools

After conducting a survey in multiple SAs, the package contains a function for estimating the density of p. When within-SA or between-SA sample sizes are small, the density estimators are biased and thus density calculations should not be interpreted literally (but as suggestive exploratory analyses).

To plot this distribution, use the `postplot` command, specifying the vector of results across SAs, as well as the sample size within SA. The user also must specify the estimation method - `crude` (histogram), `kernel` (nonparametric kernel density estimator), or `beta` (parametric Beta distribution estimator). If `kernel` is selected, the user can specify a bandwith, `bw`, or use the program default.

```
results <- c(7, 9, 14, 13, 17, 19, 12)
ss  <- 19
postplot(X=results, n=ss, method="crude", xlim=c(0,1), ylim=c(0,4.5))
postplot(X=results, n=ss, method="kernel", add=T)
postplot(X=results, n=ss, method="beta", add=T, lty=2)
legend("topleft", lty=c(1,2), lwd=2, c("Kernel", "Beta"))
```

To calculate the expected number of areas within a range, say between p_l and p_u, use the `postest` command, again specifying a method for density estimation. Standard errors are calculated using the bootstrap on samples with convergence.

With small samples, estimates and standard errors will likely be biased and are often not calculable.

```
> postest(X=results, n=ss, range=c(.35, .65), method="kernel")
 P(p in [pl,pu])        sd
       0.3692592 0.1450621
> postest(X=results, n=ss, range=c(.35, .65), method="beta")
 P(p in [pl,pu]) sd
       0.3421976 NA
Warning messages:
1: In pbeta(pu, alphahat, betahat) : NaNs produced
2: In pbeta(pl, alphahat, betahat) : NaNs produced
> postest(X=results, n=ss, range=c(.35, .65), method="kernel")
 P(p in [pl,pu])        sd
       0.3692592 0.1442439
```

4 Finite population surveys - the hypergeometric distribution

The LQAS survey design template can be extended to probability distributions other than the binomial. In finite populations, the hypergeometric distribution is the reference distribution. Specifically, we sample n individuals from a population of size N and observe X successes. We model $X \sim Hyper(N, n, pN, (1-p)N)$, where p is the population coverage, pN is the total number of successes in the population; and $(1-p)N$ is the total number of failures in the population. The classification risk equations in Equation 1 are unchanged.

To design an LQAS survey in a population of size 52, we use the `lqas` command, specifying `popsize = 52`:

```
 hyper <- lqas(pl = 0.35, pu = 0.65, alpha = 0.09, beta=0.09, popsize = 52)
```

Naturally, `popsize` is N, the size of the population from which we are sampling. When `popsize` is specified, the `lqas` function defaults to the hypergeometric distribution.

Again, to summarize the design, we type

```
> summary(hyper)
The LQAS design parameters are:

Sample Size: 15
Decision Rule: 7
```

```
Classify as high if X > d.

The true error levels for this design are:
 alpha = 0.0702
  beta = 0.0702

Design Parameters:
pl =  0.35 and pu =  0.65
alpha =  0.09 and beta =  0.09
Probabilities were calculated using the hypergeometric distribution.

Population size: 52

Effective pl: 0.3462
Effective pu: 0.6538
```

Because we are accounting for the fact that the population contains only 52 individuals, we do not need as large of a sample size as in the binomial example, which assumes an infinitely large population.

The commands for binomial designs apply to the hypergeometric design as well:

```
plot(design)
plot(design, risk=T)
plot(design, risk=T, pstar=0.65)
oc(design)
```

The risk and OC curves are now step functions, because p can only take on a discrete number of values when the population is finite. This discrete nature of the coverage probability p makes the hypergeometric calculations somewhat under-conservative in many situations. In the summary of the design, an "effective p_l and p_u" are provided (these parameters are not displayed in the binomial case, because p is not discrete in the binomial setting). The effective bound for p_l is the closest value of p that is less than or equal to p_l.

In the example above, p cannot take on the exact values $p_l = .35$ or $p_u = .65$. The effective p_l is .3462, the closest value that p can take on that is $\leq .35$. Similarly, the effective bound for p_u is .6538, the closest value that p can take on that is $\geq .65$. The distance between the effective p_l and p_u is usually greater than the distance between the specified values of p_l and p_u, which makes the design appear more conservative than it truly is. This is most problematic when the size of the population is very small or p is a rare event.

Post-estimation tools were only developed for the binomial model. However, to assess the design, we recommend calculating a binomial sampling design with the same design parameters (α, β, p_l and p_u) and applying the post-estimation tools to this single sampling plan as a sensitivity analysis.

The `lqas` function also allows the user to specify `family = poisson`, but this selection is antiquated. Historically, the Poisson distribution was used as an approximation to the binomial distribution when the sample size was large and the event of interest was rare. Calculations based on the binomial distribution are trivial using the R package, and we do not recommend using the Poisson distribution for design.

5 Cluster LQAS survey designs

The cluster LQAS survey designs implemented in this package are discussed in [2] and [3]. Cluster sampling designs are a frequently used alternative to SRS designs, where clusters (e.g. villages) are first sampled and then individuals are sampled within clusters using simple random sampling. Cluster LQAS designs assume the cluster-sampling mechanism is self-weighting, i.e. everyone in the population has an equal probability of being sampled.

Hund and Pagano (2013) propose a non-parametric sample size calculation algorithm for cluster sampling designs based on the effective sample size (ESS) [3]. To use this algorithm, the user must specify the option `ess = T`. To determine a sample size and decision rule using the `lqascluster` function, the user must again input p_l, p_u, α, and β. Now, the user must also specify the intraclass correlation coefficient ρ and either the number of clusters to sample k; the number of individuals to sample within cluster m; or a cost function that depends on m and k. The user can also specify a finite number of clusters in the population K (the [2] algorithm assumes $K = \infty$).

```
> cluster <- lqascluster(pl=.35, pu=.65, rho=.1, alpha = 0.09, beta = 0.09,
+ ess = TRUE, m = 10)
> summary(cluster)
The LQAS design parameters are:

Sample Size: 40
Decision Rule: 19

Classify as high if X > d.

The true error levels for this design are:
 alpha = 0.0772
  beta = 0.0772

Design Parameters:
```

```
pl =  0.35 and pu =  0.65
alpha =  0.09 and beta =  0.09
Probabilities were calculated assuming cluster sampling was used.
Sample 4 clusters and 10 individuals per cluster, assuming an ICC of 0.1.

#finite number of clusters in population
> finiteK <- lqascluster(pl=.35, pu=.65, rho=.1,
+ alpha = 0.09, beta = 0.09, ess = TRUE, K = 10, m = 10)
> summary(finiteK)
The LQAS design parameters are:

Sample Size: 30
Decision Rule: 14

Classify as high if X > d.

The true error levels for this design are:
 alpha = 0.0875
  beta = 0.0875

Design Parameters:
pl =  0.35 and pu =  0.65
alpha =  0.09 and beta =  0.09
Probabilities were calculated assuming cluster sampling was used.
Sample 3 out of 10 total clusters. Within each cluster,
sample 10 individuals per cluster, assuming an ICC of 0.1.
```

When specifying a cost function, the user must first design the cost function. This function should always take m as the first argument and k as the second argument. By default, the function iterates through values of k to find the minimum cost. To minimize cost iterating through values of m, specify `miter=T`.

```
> #linear cost function
> costlin <- function(m, k) { 5*k + 1*m*k }
>
> clusterlin <- lqascluster(pl=.35, pu=.65, rho=.1, alpha = .09, beta = .09,
+ ess = T, cost = costlin)
> summary(clusterlin)
The LQAS design parameters are:

Sample Size: 32
Decision Rule: 15
```

```
Classify as high if X > d.

The true error levels for this design are:
 alpha = 0.0875
  beta = 0.0875

Design Parameters:
pl =  0.35 and pu =  0.65
alpha =  0.09 and beta =  0.09
Probabilities were calculated assuming cluster sampling was used.
Sample 4 clusters and 8 individuals per cluster, assuming an ICC of 0.1.
```

In [2], the authors propose designing a cluster LQAS survey by using a convolution of beta-binomial random variables. The design specification is the same as the example above, but now the user should specify `ess = F`. This method assumes $K = \infty$, and therefore the user can no longer specify K. Choosing $\rho = .1$ and $m = 10$ results in the following design:

```
> cluster <- lqascluster(pl=.35, pu=.65, rho=.1,
+ alpha = 0.09, beta = 0.09, ess = FALSE, m = 10)
> summary(cluster)
NOTE: More than one decision rule found.
The LQAS design parameters are:

Sample Size: 50
Decision Rules: 24 25

Classify as high if X > d.

The true error levels for this design are:
 alpha = 0.05 0.073
  beta = 0.077 0.045

Design Parameters:
pl =  0.35 and pu =  0.65
alpha =  0.09 and beta =  0.09
Probabilities were calculated assuming cluster sampling was used.
Sample 5 clusters and 10 individuals per cluster, assuming an ICC of 0.1.
```

As in the example above, it is possible to have more than one decision rule that meets the specifications, due to the fact that the sample size increases in increments of m. In this case, the user must decide whether having a lower α or β error is preferable.

Post-estimation tools are not developed for cluster sampling plans. To assess the design, we recommend calculating the effective sample size and corresponding decision

rule under simple random sampling and applying the tools to this design. Formulas for the ESS are provided in [3].

5.1 Incorporating uncertainty in p^* into the design

Uncertainty in p^* can be incorporated by design, as described in [3]. The user must specify a Beta distribution for p^* or a prior for p^* along with historical data. For details on the methodology behind incorporating uncertainty in p^* and using the `lqasdiff` function, please read [3].

If the user knows the parameters for the distribution of p^*, these are specified via `params`, a vector of length 2. The parameters `dl` and `du`are deviations from the distribution of p^*. The default is `dl = 0`.

```
> knowndist <- lqasdiff(params=c(17.5, 32.5),
 dl=0, du=.3, alpha=.09, beta=.09)
> summary(knowndist)
Using an underlying beta 17.5, 32.5 distribution, the design parameters are:

Total Sample Size: 31
Decision Rule: 15

Declare no change if X <= d.

The true error levels for this design are: alpha = 0.0874 and beta = 0.0874.

Design Parameters:
Delta_l =  0 and Delta_u =  0.3
alpha =  0.09 and beta =  0.09
```

To specify a prior, along with historical data, the user specifies the prior in `params`; and the historical data in `X` and `N`. Currently, the function accommodates historical data from only one past survey.

```
> unknowndist <- lqasdiff(params=c(.1,.1), X = 35, N = 100,
dl=0, du=.3, alpha=.09, beta=.09)
> summary(unknowndist)
When 35 out of 100 failures occur at the previous timepoint, the design parameters are:

Total Sample Size: 23
Decision Rule: 11

Declare no change if X <= d.

The true error levels for this design are: alpha = 0.089 and beta = 0.0899.

Design Parameters:
```

```
Delta_l =  0 and Delta_u =  0.3
alpha =  0.09 and beta =  0.09
```

If the data were and are collected using cluster sampling, the user can specify the intraclass correlation ρ (assumed the same for historical data and current survey). The within-cluster sample size for historical data is specified in $m0$. For the current survey, the user must specify m or k, and can also specify K (same notation as above). The function currently does not accommodate cost function optimization.

6 Double Sampling

Double sampling plans collect data in two rounds of sampling. After the first round of sampling, a decision is made or the next round of sampling begins. To design a double-sampling plan, the user must specify the same parameters as in a single sampling design (α, β, p_l and p_u), as well as two additional parameters, α_1 and β_1. These parameters are defined such that $P(X_1 \leq d_1|n, p = p_u) \leq \alpha_1$ and $P(X_1 > d_1|n, p = p_l) \leq \beta_1$.

To select α_1 and β_1, we first determine whether to develop round one stopping rules for classifying areas as high or low. If you choose to stop on high classifications, choose $\beta_1 < \beta$ and $\alpha_1 = 1$; stopping on low classifications, choose $\alpha_1 < \alpha$ and $\beta_1 = 1$. Double sampling plans are designed using the `lqas` command, with the additional specified parameters and the selection `double = T`:

```
> double <- lqas(pl=0.35, pu=0.65, alpha1 = 1, beta1 = 0.05,
 alpha=0.1, beta=0.1, double=T)
> summary(double)
The LQAS design parameters are:

Sample Size 1: 3
Sample Size 2: 19

Decision Rule 1: 2
Decision Rule 2: 11

Classify as high if X1 > d1 or X1 + X2 > d2.

The true error levels for this design are: alpha = 0.0975 and beta = 0.0823.

Design Parameters:
pl =  0.35 and pu =  0.65
alpha =  0.1 and beta =  0.1
alpha1 =  1 and beta =  0.05
Probabilities were calculated using the binomial distribution.
```

Post-estimation tools are not developed for double sampling plans. However, to assess the overall design, we recommend calculating a single-sampling design with the same design parameters (α, β, p_l and p_u) and applying the post-estimation tools to this single sampling plan.

References

[1] Olives C and Pagano M. Choosing a design to fit the situation: how to improve specificity and positive predictive values using bayesian lot quality assurance sampling. *International Journal of Epidemiology*, 2013.

[2] Hedt-Gauthier B, Mitsunaga T, Hund L, Olives C, and Pagano M. An approach for incorporating clustering effects into the design of lot quality assurance sampling. March 2013.

[3] Hund L and Pagano M. Extending LQAS cluster survey designs for nutrition surveillance programs. March 2013.

[4] Hund L and Pagano M. Practical tools for designing LQAS surveys. March 2013.

[5] RStudio Inc. *shiny: Web Application Framework for R, R package*, 2013. version 0.5.0.

7 Figures

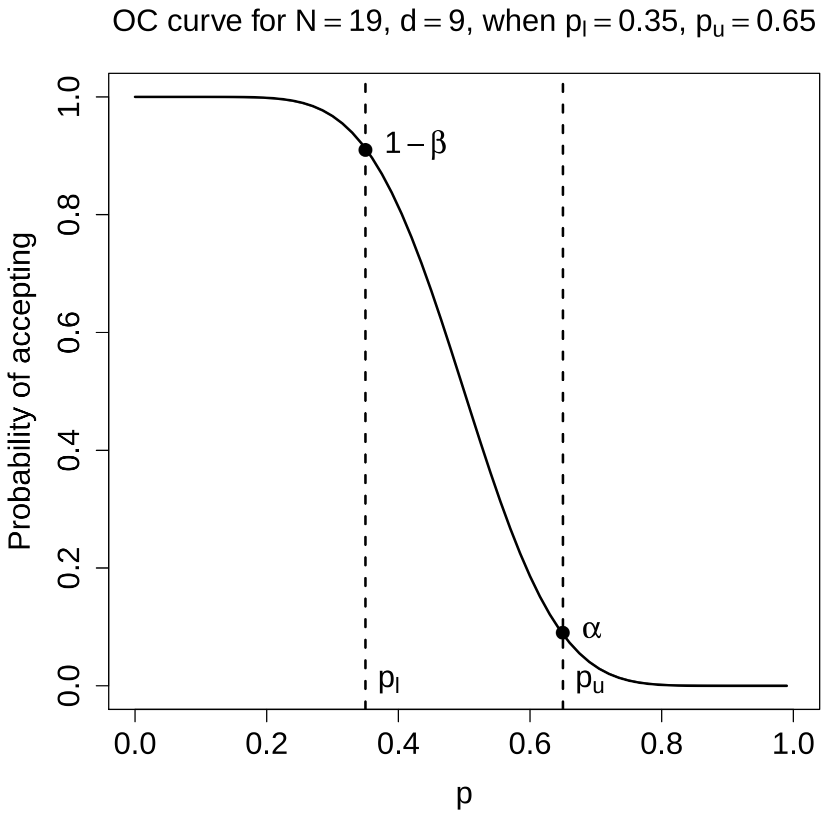

Figure 1: Plot of OC curve for survey design with $p_l = 0.35, p_u = 0.65, \alpha = 0.09, \beta = 0.09$.

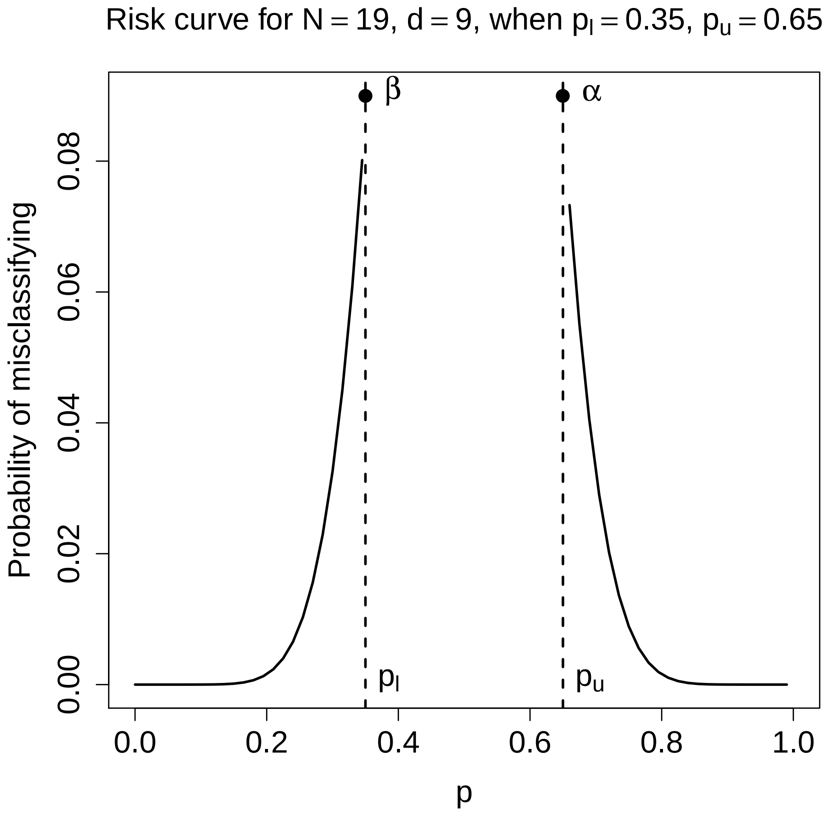

Figure 2: Plot of risk curve for survey design with $p_l = 0.35, p_u = 0.65, \alpha = 0.09, \beta = 0.09$, without specifying p^*.

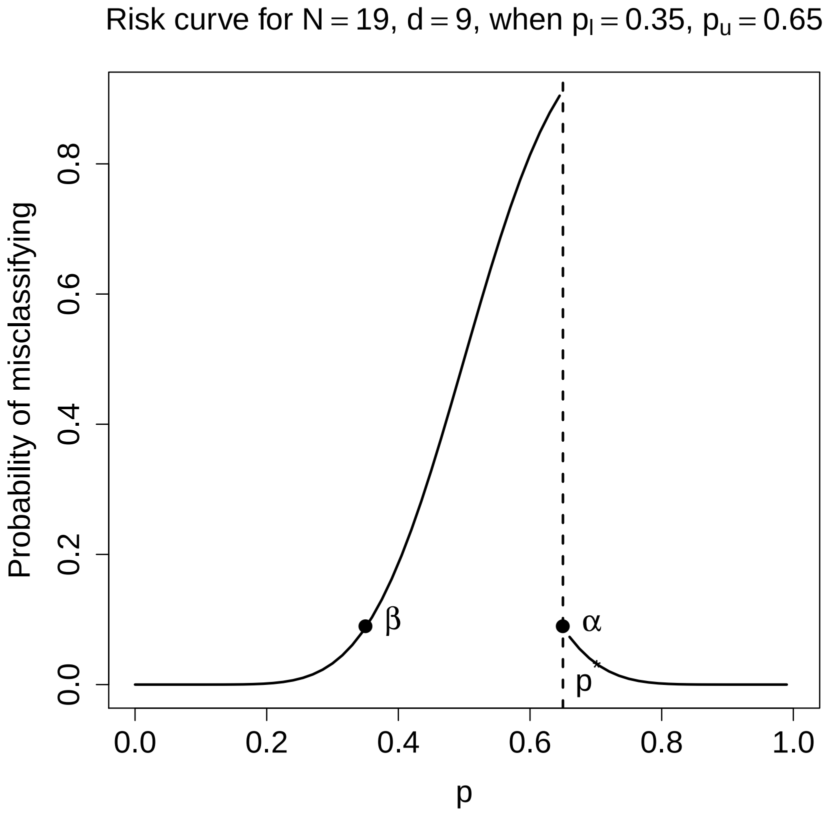

Figure 3: Plot of risk curve for survey design with $p_l = 0.35, p_u = 0.65, \alpha = 0.09, \beta = 0.09$, specifying $p^* = 0.65$.

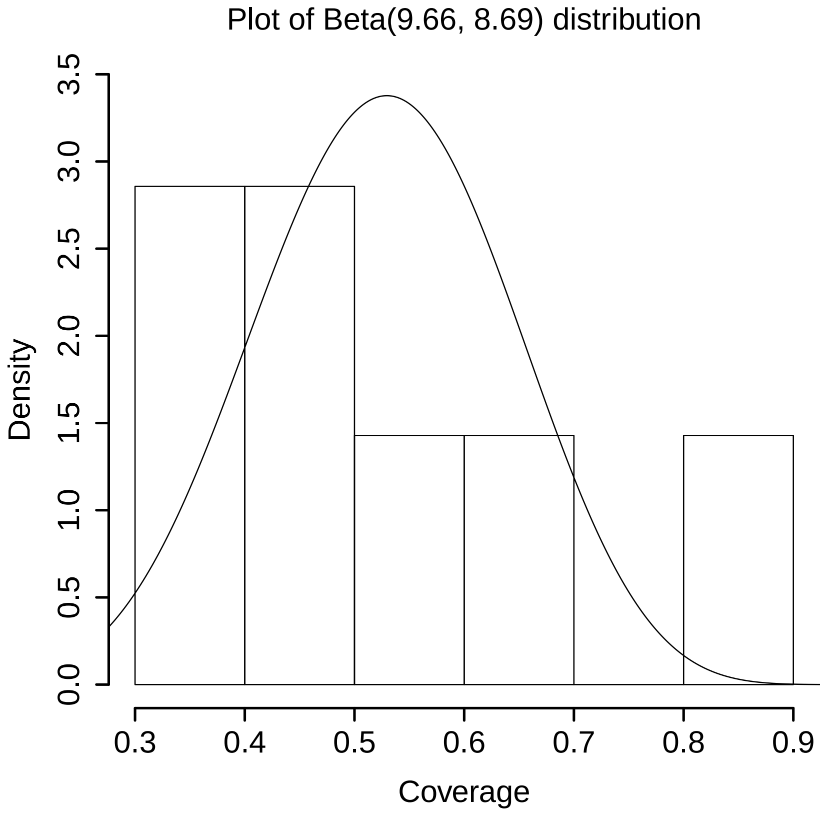

Figure 4: Prior distribution overlayed on histogram of estimated p for each SA.

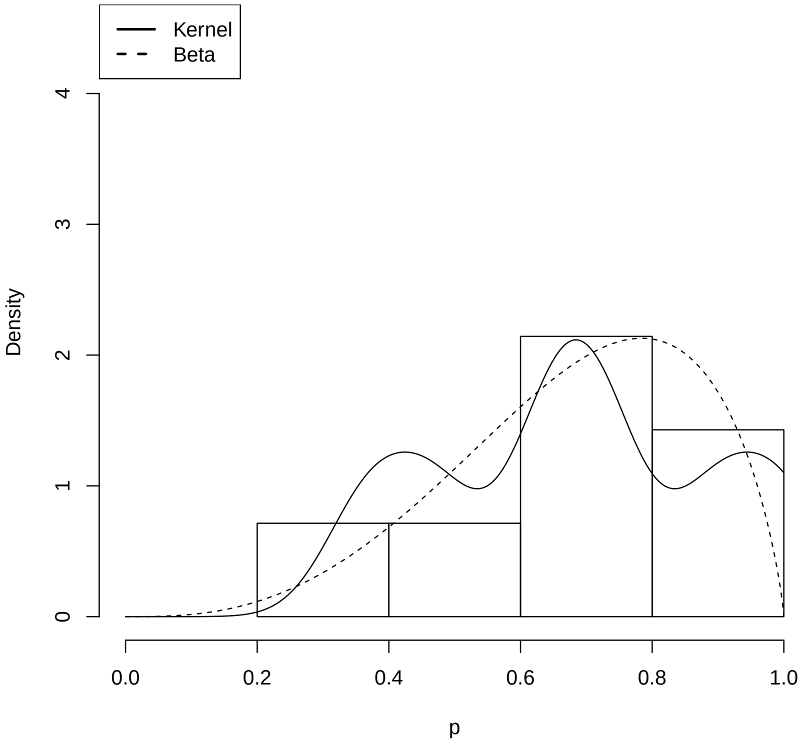

Figure 5: Posterior distribution estimates for p across SAs.
