# Supplementary material for: New tools for evaluating LQAS survey designs
Source: Emerg Themes Epidemiol. 2014 Feb 15;11:2. doi: 10.1186/1742-7622-11-2 (PMC3931287; doi:10.1186/1742-7622-11-2)
Supplement: Additional file 1 — lqasdesign R package. Additional file 2 contains the lqasdesign R package. [file 1742-7622-11-2-S1.zip › lqasdesign/html/00Index.html]

R: Design of LQAS surveys

# Design of LQAS surveys

---

## Documentation for package ‘lqasdesign’ version 1.0

- DESCRIPTION file.
- Overview of user guides and package vignettes; browse directory.

## Help Pages

|  |  |
| --- | --- |
| lqasdesign-package | LQAS survey design |
| boc | Constructs Bayesian OC curves |
| designeval | Evaluates properities of an LQAS design |
| doublesampling | LQAS double sampling survey design |
| lqas | LQAS survey design |
| lqascluster | LQAS survey design for cluster sampling surveys |
| lqasdesign | LQAS survey design |
| lqasdiff | LQAS design with uncertainty in p\* |
| lqasrisk | Calculates LQAS risks |
| makeprior | Makes a beta prior distribution for coverage. |
| oc | Returns OC probabilities for LQAS design |
| plot.doublelqas | Plot OC and Risk curves for LQAS double sampling design |
| plot.lqas | Plot OC and Risk curves for LQAS design |
| plot.prior | Plot prior distribution |
| postest | LQAS postestimation |
| postplot | LQAS postestimation desntiy plot |
| print.summary.lqaseval | Print function for LQAS design evaluation |
| summary.doublelqas | Summarize LQAS double sampling design |
| summary.lqas | Summarize LQAS design |
| summary.lqasdiff | Summary function for lqasdiff object. |
| summary.lqaseval | Summary function for LQAS design evaluation |
| summary.prior | Summarize prior information for LQAS design |
