# Supplementary material for: New tools for evaluating LQAS survey designs
Source: Emerg Themes Epidemiol. 2014 Feb 15;11:2. doi: 10.1186/1742-7622-11-2 (PMC3931287; doi:10.1186/1742-7622-11-2)
Supplement: Additional file 2 — Appendices. Appendix A contains a description of the Beta distribution; Appendix B contains a description of the web-based R application; Appendix C contains complete R code for reproducing the analysis in the manuscript; and Appendix D contain a simulation study assessing properties of the density estimators. [file 1742-7622-11-2-S2.pdf]

Appendix to "New tools for evaluating LQAS survey designs"

Lauren Hund

October 31, 2013

Appendix A - The beta distribution

For a random variable p_i that can take values between 0 and 1, if $p_i \sim Beta(a, b)$, then the following are true:

$$E(p_i) = \frac{a}{a+b}$$

$$Var(p_i) = \frac{ab}{(a+b)^2(a+b+1)}$$

$$\rho = \frac{1}{a+b+1}$$

Appendix B - Web-based application

The web-based application can also reproduce many analyses in the paper and is easier to use for those not familiar with R. To access the suvey design application, type `designsurvey()` into the R prompt, after installing the R package.

To install the R package, use the following steps:

1. Save the zip file on your computer.
2. Install and open R.
3. Under the packages menu, select "install package from local zip file" and choose the `lqasdesign` R package zip file.
4. Type `library(lqasdesign)` in the R command line and hit enter.

To select a beta-prior for use in the survey evaluation, type `surveyprior()` into the R command prompt to access a web-application for prior selection.

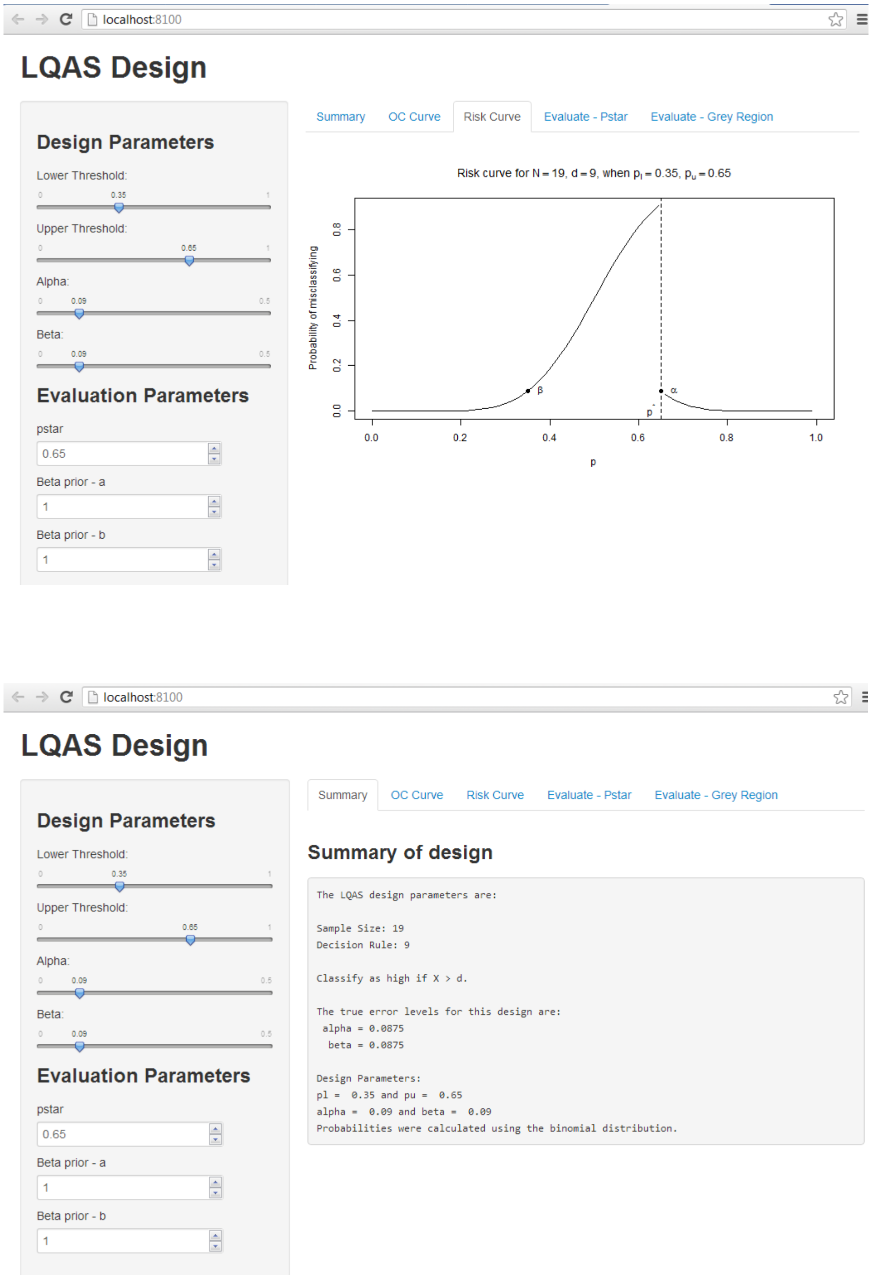

Figure 1: Screen-shots from web-based application for survey design.

Appendix C - R code for replicating the data analysis

R code for reproducing analysis:

```
library(lqasdesign)

### make survey design ###
design <- lqas(pl = .35, pu = .65, alpha = .09, beta=.09)
summary(design)


### plot OC and risk curves ###
plot(design)
plot(design, risk=T)
plot(design, risk=T, pstar=.65)


### construct prior distributions ###
results <- c(7, 7, 12, 9, 11, 16, 8)
ss        <- 19

prior  <- makeprior(X=results, n=ss)
prior2 <- makeprior(mean = prior$mean + .15, sd = prior$sd)
prior3  <- makeprior(mean = prior$mean + .15, sd = prior$sd/2)
prior4  <- makeprior(mean = prior$mean + .15, sd = 1.25*prior$sd)

summary(prior)
summary(prior2)
summary(prior3)
summary(prior4)

plot(prior, ylim=c(0, 5), xlim=c(0,1), lwd=2, cex.axis=1.5, cex.lab=1.5, main="")
plot(prior2, add=T,lty=2, lwd=2)
plot(prior3, add=T, lty=3, lwd=2)
plot(prior4, add=T, lty=4, lwd=2)
legend("topleft", c("B(9.6, 8.7)", "B(4.3, 2.1)",
"B(19.4, 9.3)", "B(2.5, 1.2)"), lty=c(1,2,3,4), lwd=2, cex=1.2)


### evaluate the design with respect to different prior distributions ###
eval0 <- designeval(n=19, d=9, pstar=.65, prior=c(1,1))
eval1 <- designeval(n=19, d=9, pstar=.65, prior=c(prior$alpha, prior$beta))
eval2 <- designeval(n=19, d=9, pstar=.65, prior=c(prior2$alpha, prior2$beta))
eval3 <- designeval(n=19, d=9, pstar=.65, prior=c(prior3$alpha, prior3$beta))
```

```
eval4 <- designeval(n=19, d=9, pstar=.65, prior=c(prior4$alpha, prior4$beta))

summary(eval0)
summary(eval1)
summary(eval2)
summary(eval3)
summary(eval4)

eval0 <- designeval(n=19, d=9, pstar=.35, prior=c(1,1))
eval1 <- designeval(n=19, d=9, pstar=.35, prior=c(prior$alpha, prior$beta))
eval2 <- designeval(n=19, d=9, pstar=.35, prior=c(prior2$alpha, prior2$beta))
eval3 <- designeval(n=19, d=9, pstar=.35, prior=c(prior3$alpha, prior3$beta))
eval4 <- designeval(n=19, d=9, pstar=.35, prior=c(prior4$alpha, prior4$beta))

summary(eval0)
summary(eval1)
summary(eval2)
summary(eval3)
summary(eval4)

eval0 <- designeval(n=19, d=9, pl=.35, pu=.65, prior=c(1,1))
eval1 <- designeval(n=19, d=9, pl=.35, pu=.65, prior=c(prior$alpha, prior$beta))
eval2 <- designeval(n=19, d=9, pl=.35, pu=.65, prior=c(prior2$alpha, prior2$beta))
eval3 <- designeval(n=19, d=9, pl=.35, pu=.65, prior=c(prior3$alpha, prior3$beta))
eval4 <- designeval(n=19, d=9, pl=.35, pu=.65, prior=c(prior4$alpha, prior4$beta))

summary(eval0)
summary(eval1)
summary(eval2)
summary(eval3)
summary(eval4)


### conduct posterior distribution analysis ###
results <- c(7, 9, 14, 13, 17, 19, 12)
ss  <- 19
postplot(X=results, n=ss, method="crude", xlim=c(0,1), ylim=c(0,2.5))
postplot(X=results, n=ss, method="kernel", add=T, lwd=2)
postplot(X=results, n=ss, method="beta", add=T, lty=2, lwd=2)
legend("topleft", lty=c(1,2), lwd=2, c("Kernel", "Beta"), cex=1.3)

postest(X=results, n=ss, range=c(.35, .65), method="kernel")
postest(X=results, n=ss, range=c(.35, .65), method="beta")
postest(X=results, n=ss, range=c(.35, .65), method="crude")
```

Appendix D - Simulation study assessing finite sample bias in density estimators

Finite sample properties of the density estimators are examined as a function of both the SA sample size and the number of SAs sampled. Consider scenarios in which 10, 20, 50, and 100 SAs are included in the survey; and, within each SA, consider LQAS sample sizes of $n = 20, 50$, and 100. I simulate data using two different distributions for $\pi()$: (1) a bimodal distribution that is an equally weighted mixture of two beta random variables centered at .3 and .7, with intraclass correlation $\rho = .05$, $.5B(5.7, 13.3) + .5B(13.3, 5.7)$; and (2) a beta distribution centered at .5. with $\rho = .1$, $B(4.5, 4.5)$. These two distributions are plotted in Figure 2. I simulate LQAS-type data from these distributions, estimate the underlying density, and then use this distribution to estimate $P(p_l < p_i < p_u)$, the probably of an SA having prevalence in the grey region. The simulations are repeated for two scenarios: $p_l = .35, p_u = .65$ and $p_l = .65, p_u = .85$. I average over 1000 simulations to obtain the results. To calculate standard errors, I use 200 bootstrap replications. Results are shown in Table 1 and 2.

The parametric beta density estimator has the smallest bias and variance when the model is correct, as expected. When the model is not correct, the parametric model is very biased. Further, standard errors often cannot be estimated using the bootstrap for the beta model in small sample sizes ($n = 20, 50$). The simulation results are based on the simulation samples with valid standard error estimates.

The nonparametric histogram estimator performs relatively well in terms of bias, though has the highest variance. When the within-SA sample size is small ($n = 20$), the histogram estimator can be severely biased. The kernel density estimator is also biased in small samples, with bias decreasing with the sample size. The within-SA sample size n drives the degree of bias more than the number of SAs sampled. No single density estimator consistently outperforms the others. In large sample sizes, the kernel or histogram estimators are preferable to the parametric beta distribution, to avoid bias due model misspecification. In practice, when sample sizes are small, estimating all of the densities and comparing differences between the estimated densities can give an idea of the potential for bias in the estimates.

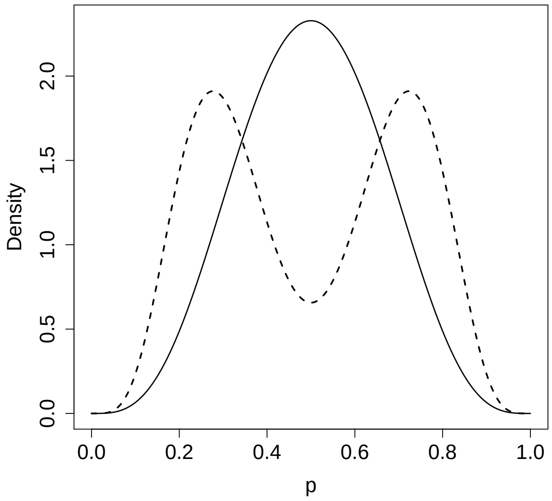

Figure 2: Data-generating densities in the simulation study.

Unimodal $B(4.5, 4.5)$											
		True	Parametric Beta			Nonparametric Histogram			Non-parametric Kernel		
SAs	n	p_g	$E(\hat{p}_g)$	$E[se(\hat{p}_g)]$	$sd(\hat{p}_g)$	$E(\hat{p}_g)$	$E[se(\hat{p}_g)]$	$sd(\hat{p}_g)$	$E(\hat{p}_g)$	$E[se(\hat{p}_g)]$	$sd(\hat{p}_g)$
10	20	.63	.66	.12	.16	.62	.14	.16	.53	.13	.14
10	50	.63	.65	.11	.13	.59	.15	.16	.58	.13	.13
10	100	.63	.66	.11	.13	.63	.14	.16	.61	.13	.14
20	20	.63	.65	.10	.12	.62	.11	.11	.54	.10	.10
20	50	.63	.64	.09	.09	.59	.11	.11	.58	.10	.10
20	100	.63	.65	.08	.09	.63	.10	.11	.61	.09	.10
50	20	.63	.64	.07	.08	.62	.07	.07	.54	.06	.06
50	50	.63	.63	.06	.06	.58	.07	.07	.58	.06	.06
50	100	.63	.63	.05	.05	.62	.07	.07	.61	.06	.06
100	20	.63	.63	.05	.05	.61	.05	.05	.54	.05	.05
100	50	.63	.63	.04	.04	.59	.05	.05	.59	.05	.05
100	100	.63	.63	.04	.04	.62	.05	.05	.61	.05	.05
Mixture of Betas											
		True	Parametric Beta			Nonparametric Histogram			Non-parametric Kernel		
SAs	n	p_g	$E(\hat{p}_g)$	$E[se(\hat{p}_g)]$	$sd(\hat{p}_g)$	$E(\hat{p}_g)$	$E[se(\hat{p}_g)]$	$sd(\hat{p}_g)$	$E(\hat{p}_g)$	$E[se(\hat{p}_g)]$	$sd(\hat{p}_g)$
10	20	.30	.45	.10	.12	.40	.15	.16	.35	.12	.12
10	50	.30	.45	.09	.09	.32	.14	.14	.34	.11	.11
10	100	.30	.44	.08	.08	.33	.14	.14	.34	.11	.11
20	20	.30	.45	.08	.08	.40	.11	.11	.34	.09	.09
20	50	.30	.44	.06	.06	.32	.10	.10	.33	.09	.08
20	100	.30	.44	.05	.06	.32	.10	.10	.32	.08	.08
50	20	.30	.44	.05	.05	.40	.07	.07	.33	.06	.06
50	50	.30	.44	.04	.04	.32	.06	.07	.32	.06	.06
50	100	.30	.44	.03	.03	.32	.07	.07	.32	.06	.06
100	20	.30	.44	.03	.03	.40	.05	.05	.34	.04	.04
100	50	.30	.44	.03	.03	.32	.05	.05	.32	.04	.04
100	100	.30	.43	.02	.02	.32	.05	.05	.31	.04	.04

Table 1: Evaluating finite sample bias for density estimators for two different underlying densities when $p_l = .35, p_u = .65$. The probably of an SA having true prevalence in the grey region is $p_g = P(p_l < p_i < p_u)$

Unimodal $B(4.5, 4.5)$											
		True	Parametric Beta			Nonparametric Histogram			Non-parametric Kernel		
SAs	n	p_g	$E(\hat{p}_g)$	$E[se(\hat{p}_g)]$	$sd(\hat{p}_g)$	$E(\hat{p}_g)$	$E[se(\hat{p}_g)]$	$sd(\hat{p}_g)$	$E(\hat{p}_g)$	$E[se(\hat{p}_g)]$	$sd(\hat{p}_g)$
10	20	.18	.15	.09	.10	.25	.12	.14	.19	.09	.10
10	50	.18	.16	.09	.10	.19	.11	.12	.19	.09	.10
10	100	.18	.16	.08	.09	.20	.11	.13	.18	.09	.11
20	20	.18	.16	.07	.08	.25	.09	.09	.20	.07	.07
20	50	.18	.17	.06	.07	.20	.08	.09	.20	.07	.08
20	100	.18	.17	.06	.06	.19	.08	.09	.18	.07	.08
50	20	.18	.17	.05	.05	.25	.06	.06	.20	.05	.05
50	50	.18	.18	.04	.04	.19	.05	.05	.19	.05	.05
50	100	.18	.17	.04	.04	.20	.06	.06	.19	.05	.05
100	20	.18	.17	.04	.03	.25	.04	.04	.20	.04	.04
100	50	.18	.17	.03	.03	.19	.04	.04	.19	.04	.04
100	100	.18	.18	.03	.03	.19	.04	.04	.19	.04	.04
Mixture of Betas											
		True	Parametric Beta			Nonparametric Histogram			Non-parametric Kernel		
SAs	n	p_g	$E(\hat{p}_g)$	$E[se(\hat{p}_g)]$	$sd(\hat{p}_g)$	$E(\hat{p}_g)$	$E[se(\hat{p}_g)]$	$sd(\hat{p}_g)$	$E(\hat{p}_g)$	$E[se(\hat{p}_g)]$	$sd(\hat{p}_g)$
10	20	.32	.21	.08	.09	.31	.13	.15	.24	.10	.11
10	50	.32	.21	.08	.08	.29	.13	.14	.26	.11	.11
10	100	.32	.22	.08	.08	.32	.14	.15	.28	.11	.12
20	20	.32	.22	.06	.06	.32	.10	.10	.25	.08	.08
20	50	.33	.22	.06	.05	.28	.10	.10	.27	.08	.08
20	100	.32	.22	.05	.05	.32	.10	.10	.29	.08	.09
50	20	.32	.22	.04	.03	.31	.06	.07	.25	.05	.05
50	50	.32	.22	.03	.03	.29	.06	.07	.28	.06	.06
50	100	.32	.22	.03	.03	.32	.07	.07	.30	.06	.06
100	20	.32	.22	.02	.02	.31	.05	.04	.26	.04	.04
100	50	.32	.22	.02	.02	.29	.05	.04	.29	.04	.04
100	100	.32	.22	.02	.02	.32	.05	.05	.31	.04	.04

Table 2: Evaluating finite sample bias for density estimators for two different underlying densities when $p_l = .6, p_u = .85$. The probably of an SA having true prevalence in the grey region is $p_g = P(p_l < p_i < p_u)$
